# Cough in Idiopathic Pulmonary Fibrosis

**DOI:** 10.3389/fresc.2021.751798

**Published:** 2021-10-18

**Authors:** Jennifer Mann, Nicole S. L. Goh, Anne E. Holland, Yet Hong Khor

**Affiliations:** ^1^Department of Respiratory and Sleep Medicine, Austin Health, Melbourne, VIC, Australia; ^2^Institute for Breathing and Sleep, Melbourne, VIC, Australia; ^3^Department of Medicine (Austin Health), University of Melbourne, Melbourne, VIC, Australia; ^4^Department of Physiotherapy, Alfred Health, Melbourne, VIC, Australia; ^5^Central Clinical School, Monash University, Melbourne, VIC, Australia

**Keywords:** cough, idiopathic pulmonary fibrosis, interstitial lung disease, quality of life, patient reported outcome (PRO) measures, prognosis

## Abstract

Chronic cough is experienced by most patients with idiopathic pulmonary fibrosis (IPF). It is often the first symptom and is associated with reduced quality of life, increased rates of depression and anxiety, more severe physiological impairment, and disease progression. Although not fully understood, recent gains in understanding the pathophysiology of chronic cough in IPF have been made. The pathophysiology is likely multifactorial and includes alterations in mucous production and clearance, architectural distortion, and increased cough reflex sensitivity, suggesting a role for targeted therapies and multidisciplinary treatment. Modifiable comorbidities can also induce cough in patients with IPF. There is a renewed emphasis on measuring cough in IPF, with clinical trials of novel and repurposed therapies for chronic cough emerging in this population. This review provides an update on the clinical characteristics, pathophysiology, and measurement of chronic cough in patients with IPF and summarizes recent developments in non-pharmacological and pharmacological therapies.

## Introduction

Idiopathic pulmonary fibrosis (IPF) is a chronic fibrosing idiopathic interstitial pneumonia, most prevalent after the 6th decade of life. It is associated with significant morbidity and mortality, with progressive worsening of respiratory symptoms over time. Cough is a common symptom, often occurring years prior to diagnosis, contributing to poor prognosis and reduced quality of life. The severity of cough encompasses its frequency, intensity, and impact to patients' lives (1). Cough frequency is an objective measurable index, which is commonly expressed as absolute cough counts and number of coughs per hour. In the absence of a gold standard measuring cough intensity, or the force of cough, it has been assessed objectively using physiological measures such as expiratory flow in research settings, and subjectively using patient reported instruments for its impact. Along with accurate assessment of cough severity, improved understanding of the pathophysiological mechanisms and contributors to cough, and development of evidence-based management are vital to improve quality of life in patients with IPF.

## Cough Characteristics

### Epidemiology and Demographics

Cough and dyspnoea are the two most common symptoms described in patients with IPF, with up to 80% experiencing chronic cough (2). Cough is often the first symptom and may precede diagnosis by months to years. Female gender and never-smoking status are associated with increased cough frequency in patients with IPF measured objectively by 24-h cough recording (3). Unexpectedly, smoking history is the strongest predictor of cough in IPF, with current or ex-smokers less likely to report cough (2). Smokers have reduced cough counts in response to capsaicin provocation when compared to non-smokers. Possible mechanisms include nicotine-induced inhibition of C fibers, changes in mucociliary clearance or desensitization of cough receptors in airway epithelium (4).

Cough is more prevalent in patients with advanced IPF (2). Increased cough frequency measured by mean coughs per hour have been associated with reduced exercise tolerance and higher serum levels of Krebs von den Lungen 6 molecule (5), a marker of alveolar epithelial cell injury currently under evaluation as a prognostic biomarker in IPF. In a cohort of 242 patients with IPF, the presence of cough was an independent predictor of disease progression, defined as a 15% decline in the diffusing capacity of the lungs for carbon monoxide, a 10% decline in forced vital capacity, lung transplantation, or all-cause mortality (2). Despite this, further evidence is required to conclusively demonstrate the relationship between cough and disease progression in patients with IPF.

### Cough Description and Impacts

Cough due to IPF is typically described as dry or non-productive and refractory to antitussive therapy. A minority of patients have a chesty cough from the onset of disease. The severity of cough in IPF is greater than in other types of interstitial lung disease (ILD) (6–8). The intensity of cough increases significantly before death, although less markedly than for dyspnoea (9). Using 24-h objective cough frequency monitoring, diurnal variation is observed in IPF-related cough (10). The median daytime cough counts are 14.6–25 per hour compared to the median night-time cough counts of 1.9–9 per hour, although there are wide interindividual variations (10, 11). This diurnal variation is possibly related to the inhibition of cough by cortical pathways at night.

Patients identify cough as a key symptom associated with physical and emotional distress, with a more limited appreciation by health care professionals (12). Physical symptoms associated with cough include chest pain, light-headedness, cough-related syncope, hoarse voice, incontinence, and sleep disturbance (1, 13). Severe cases may cause vomiting and even rib fractures (14). Some patients experience cough evoking spells of severe breathlessness and anxiety. The psychosocial effects of IPF-related cough are generally under-recognized (12), although they can cause disruption to work and social activities, tensions in relationships, and embarrassment. Management of IPF-related cough often involves trying different therapies with limited effects, which can be frustrating for patients and clinicians (15). Similarly, caregivers for patients with IPF report specific concerns regarding the need for guidance around symptom monitoring for cough (16). Thus, it is essential to assess for the presence and impact of cough in patients with IPF to enable focused intervention to address patients' needs.

## Pathophysiology

**Figure 1:**
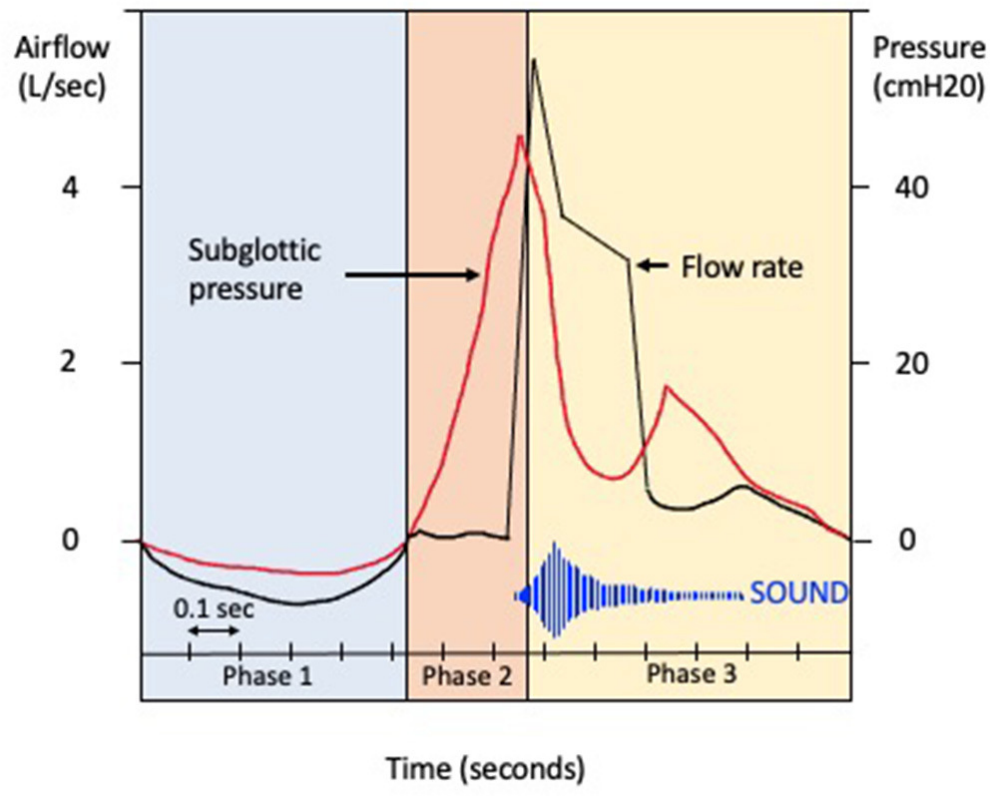
Phases of cough. Schematic representation of the changes in airflow (liters per second) and subglottic pressure (centimeter of water) during the phases of cough. Phase 1. The glottis opens on activation of the cough reflex, and a deep breath is inhaled (inspiratory phase). Phase 2. The glottis then closes and expiratory muscles forcibly contract (compressive phase), with a transient increase in intrathoracic pressure. Phase 3. The glottis opens with rapid airflow, causing oscillation of the bronchial tissues (expulsive phase).

**Figure 2:**
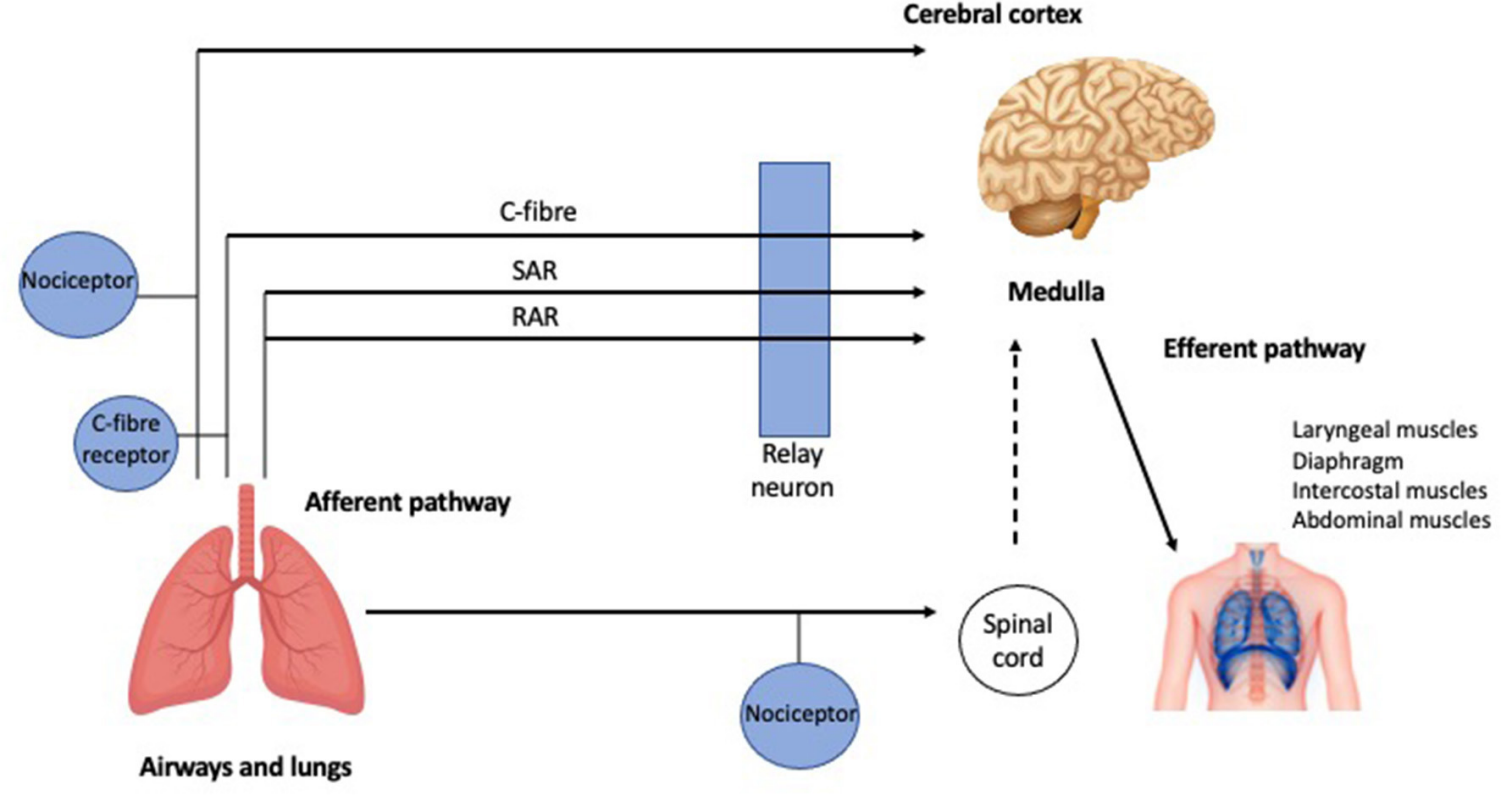
The cough reflex. Schematic representation of the afferent and efferent pathways of the cough reflex (further detail in the text). RAR, Rapidly adapting receptors; SAR, slowly adapting stretch receptors [adapted from Mazzone (19) with permission from Elsevier].

Coughing is an important defensive reflex that enhances clearance of secretions and particulates from airways and prevents aspiration of foreign materials. It is now accepted that there is also an element of behavioral voluntary control (17). There are three phases of a cough (Figure 1): the initial deep inspiration, compression of intrathoracic air due to forced exhalation against a closed glottis, and the final expiration with glottic release (18). The cough reflex (Figure 2) is triggered *via* rapidly adapting receptors, slowly adapting stretch receptors, and c-fibers in the afferent pathway that conduct mechanoreceptor and chemoreceptor stimuli to the medullary control center by branches of the vagus nerve (20). Cough receptors are most concentrated in upper airways, but also present in distal airways and lung parenchyma (21). Efferent signals are carried to the diaphragm *via* the phrenic nerves, to the abdominal muscles *via* the spinal motor nerves, and to the larynx *via* the laryngeal branches of the vagus nerve (21). While the pathophysiology of chronic cough in IPF is not fully understood, the most accepted theories propose multifactorial causation, including mechanical, chemical, and biological factors.

### Mechanical Distortion

Hallmarks of lung parenchymal damage in IPF include architectural distortion with volume loss and traction bronchiectasis. There are a number of theories as to how mechanical distortion might result in the development of cough in IPF, although none are proven. Mechanical stress from fibrotic tissue “pulling apart” the small airways may contribute to cough in IPF by sensitizing rapidly adapting receptors within the peripheral airways (22), thereby lowering the cough threshold. It has also been postulated that the loss of healthy lung tissue may destroy inhibitory nerves involved in the cough reflex (23). External percussion of the chest wall has been shown to induce cough in patients with IPF, particularly when lung bases, the part most affected in IPF, is stimulated (24). Total cough count in response to mechanical stimulation correlates with subjective measures of cough severity, including the Visual analog scale (VAS), Leicester Cough Questionnaire (LCQ), and Cough Symptom Score (24). Similarly, vibration caused by talking may increase mechanical stimulation of sensory receptors in this population (24). There is interest in cough as an additional source of mechanical stretch, contributing to a pro-fibrotic feedback loop. Mechanical tissue stretch has been shown to activate transforming growth factor-beta 1, a key pro-fibrotic cytokine that has been implicated in the development of pulmonary fibrosis in animal models (25). In other words, cough may beget cough, becoming a self-perpetuating vicious cycle. If proven, this may explain why cough can be associated with disease progression in IPF (2). Cough as a driver of disease progression (26) requires exploration, focusing on mechanisms of stretch injury to alveoli at the lung periphery (27).

### Enhanced Cough Reflex

Patients with IPF demonstrate a heightened cough reflex response to capsaicin (28) and substance *P* with an increased urge to cough, compared to healthy individuals (29). This response is reproducible and likely related to the underlying disease process (29), although the exact mechanism remains unclear. The absence of change in the capsaicin-induced cough sensitivity in simulated lung volume restriction in healthy individuals by chest strapping supports the presence of altered cough reflex in patients with IPF, independent of ventilatory defects (28). Inflammatory mediators activate cough receptors acutely, but recent progress in the pathogenesis of chronic cough suggests an additional role in neuroplasticity through the production and release of neurotrophins (17). Neurotrophins are proteins that induce the survival, development, and function of different groups of sensory nerves, which can alter and augment cough reflex sensitivity (28). Immunohistochemical studies have demonstrated enhanced expression of the neurotrophins nerve growth factor (NGF) and brain-derived neurotrophic factor in lungs affected by IPF compared to controls. Increased levels of NGF have also been found in induced sputum samples from patients with IPF compared to controls (22), with upregulation of messenger ribonucleic acid for NGF in alveolar macrophages sampled with bronchoalveolar lavage (30). Patients with IPF have sputum eosinophilia and increased sputum eosinophilic cationic protein concentration compared to normal controls (31), raising the possibility that eosinophils may modulate the cough reflex by increasing the presence of G protein-coupled receptor and substance P in human airway nerves (32).

### Change in Mucous Production

While the exact pathogenesis of IPF is yet to be elucidated, genetic variation is an established risk factor for its development. A common variant in the promoter region of the mucin 5B (MUC5B) gene, the most well-validated genetic risk factor for IPF (33–35), increases production of MUC5B, an airway mucin. The presence of the minor allele of the MUC5B promoter polymorphism (rs35705950) correlates with a clinical phenotype of IPF characterized by more severe cough (36), which is independent of age, gender, and secondary causes of cough.

## Role of Comorbidities

IPF-related comorbidities and other health conditions can be important contributors to cough in this population and are therefore key targets for treatment. Table 1 summarizes common remedial conditions associated with chronic cough and conditions not to be missed. Careful evaluation is required to diagnose and address these comorbidities. In particular, patients with IPF are at increased risk for lung cancer with adverse impacts on survival (51). Gastroesophageal reflux (GOR) and obstructive sleep apnoea (OSA) are two common comorbidities associated with cough and are discussed in more detail below.

**Table 1:** Comorbid conditions associated with chronic cough in patients with IPF.

**System**	**Diagnosis**	**Proposed mechanisms of cough**	**Prevalence in IPF**
Respiratory and sleep	Infection: tuberculosis, pertussis, lung abscess, protracted bacterial bronchitis	Excess mucous production, loss of ciliary structure, airway inflammation	NA^*^
	Chronic obstructive pulmonary disease, bronchiectasis	Excess mucous production, C-fiber nerve activity	6–67% (37)
	Asthma	Bronchial hyperreactivity	8.5–18.6% (38)^†^
	Lung cancer	C-fiber nerve activity	10–30% (39)
	Obstructive sleep apnoea	Upper airway inflammation and injury due to airway obstruction	22–90% (40)
Ear nose and throat	Earwax or foreign body	Stimulation of branch of the vagal nerve innervating external auditory canal	NA
	Chronic sinusitis	Direct irritation of the vocal cords, sensitization of the cough reflex	NA
	Vocal cord dysfunction	Paradoxical vocal cord movement and glottis closure with airway narrowing	NA
Gastrointestinal	Gastro-esophageal reflux disease	Microaspiration and direct irritation, sensitization of the cough reflex due to activation of vagal nerve endings in the esophagus	87–94% (41–44)^‡^
Cardiovascular	Left ventricular failure	Pulmonary c-fibers activated by pulmonary venous congestion and oedema (45)	4–26% (37)
	Arrhythmia	Mediators of cough (bradykinin and substance *P*) accumulate in the upper airway	NA^§^
Drugs/toxins	Angiotensin-converting enzyme inhibitor use	Mediators of cough (bradykinin and substance *P*) accumulate in the upper airway	NA^¶^

*IPF, idiopathic pulmonary fibrosis; NA, not available; TB, tuberculosis*.

* *Incidence of TB 6.3% (46) in an observational study of 143 patients in South Korea*.

† *Asthma diagnosed in the year prior to IPF diagnosis*.

‡ *GOR measured by ambulatory pH monitoring rather than impedance*.

§ *In a retrospective study of patients who underwent lung transplantation for IPF, the incidence of atrial arrhythmia was 25.4% in 366 patients (47)*.

¶ *Note in the ASCEND (48) and CAPACITY (49) studies 111 of 624 patients with IPF were on an Angiotensin-converting enzyme (ACE) inhibitor at randomization (50)*.

### Gastroesophageal Reflux

The role of GOR in IPF remains unsettled, with inconclusive results on therapeutic effects of antacid medication and anti-reflux surgery on progression-free survival, quality of life, and all-cause mortality in this population (41, 52). As distinct from acid reflux, the presence of hiatus hernia has been more clearly associated with disease progression and mortality (53). GOR is a well-accepted contributing factor to chronic cough in the general population and is included in chronic cough assessment and treatment algorithms. Patients with GOR-associated cough may not report typical symptoms such as heartburn, which are mediated by the spinal pathways, and not the vagus nerve.

The prevalence of GOR is higher in IPF than in other chronic lung diseases (42, 54, 55) and the general population (56). Eighty-seven to ninety-four percent of patients with IPF have evidence of reflux measured by ambulatory pH monitoring, even in the absence of symptoms (43) and despite treatment with acid-suppressive therapies. Cough frequency does not improve despite verifiable reductions in esophageal acid exposure, as non-acid reflux may remain or worsen with antacid medication (44). In addition to causing cough, reflux has been proposed to drive the perpetuation of fibrosis with inflammation and injury secondary to recurrent micro-aspiration. In animal models of IPF, pulmonary fibrosis was induced by direct instillation of acid into the airways (57).

Substance *P* and mast cell-tryptase are both elevated in the sputum of patients with chronic cough and comorbid GOR, which inversely correlate with cough threshold (58). In patients with IPF, salivary pepsin, a marker of proximal GOR, is higher in those with daily cough compared to those with infrequent cough (59). Both acid and non-acid events contribute to lung inflammation and fibrosis (60), suggesting that even in those patients on antacid medication, non-acid events may perpetuate the cycle of inflammation. Additionally, the long-term use of proton pump inhibitors has been associated with higher rates of respiratory infection and other side effects, including fracture risk and nutritional deficiencies (52, 61, 62). Thus, in the absence of proven or symptomatic reflux, empirical use of antacid medication is not currently recommended (63).

### Obstructive Sleep Apnoea

Sleep-disordered breathing, including OSA and sleep-related hypoxaemia, are increasingly recognized as important clinical features in IPF. The prevalence of OSA increases with age, which overlaps with that of IPF, and severity plateaus around the age of 65 in patients with stable body mass index (64). The exact prevalence of OSA (defined as an apnoea-hypopnoea index ≥ 5) in IPF is difficult to establish, with a wide range of values [22–90% (40)] due to the small number of participants in many studies. Most studies report higher apnoea hypopnoea index in patients with IPF than that of the general population (65).

The relationship between sleep-disordered breathing and cough in IPF has only been investigated in a limited number of studies. OSA is a recognized remedial cause of chronic cough in the non-IPF population (66) and responds to treatment with continuous positive airway pressure (CPAP). There are no studies that measure the effect of CPAP on cough in patients with IPF using objective cough monitors or cough-specific questionnaires. Rather, in a study of 12 patients with newly diagnosed IPF and moderate-to-severe OSA, 48% of patients complained of nocturnal cough during treatment with CPAP (67). Some patients with IPF are intolerant of CPAP and experience claustrophobia associated with a rapid and shallow breathing pattern and cough (67, 68). Additionally, cough can be exacerbated by dry air during CPAP initiation, which may be addressed using heated humidification. CPAP has been shown to improve sleep and quality of life in patients with IPF and comorbid OSA (69) and there may be a role for a trial of CPAP to address cough in patients with nocturnal cough. An acclimatization period with CPAP at lower pressures and intense clinician follow-up may be of benefit.

## Evaluation

Chronic cough is a significant challenge for the treating clinician and healthcare team, particularly in IPF where it is often refractory to antitussive therapy. A systematic approach to evaluation, starting with clinical assessment for cough characteristics, comorbidities, and non-IPF causes of cough is necessary. While well-accepted in clinical research and multidisciplinary cough clinics, cough measurement has not yet been incorporated into routine clinical practice. Nevertheless, it is an important step in assessing severity, response to treatment, and as a communication tool for patients and caregivers.

### Clinical Assessment

In patients with IPF who present with a troublesome cough, a timely and thorough history-taking, clinical examination, basic blood workup, chest radiograph and lung function testing is recommended. An attempt should be made to distinguish IPF-related cough from non-IPF-related cough (63). A sudden worsening of symptoms or significant deterioration in forced vital capacity (FVC) or transfer factor for carbon monoxide (TL_CO_) on lung function testing may prompt further investigation with computed tomography of the chest to assess for acute exacerbation, infection, or disease progression. Red flag symptoms for lung cancer include haemoptysis, dysphonia or dysphagia, and weight loss. Relevant comorbidities (Table 1) should be actively considered, and treatment trials or further clinical investigations should be pursued according to clinical assessment. Some examples include nasolaryngoscopy for sinus and upper airway pathology, polysomnography for sleep-disordered breathing and impedance testing for silent GOR.

### Objective Cough Measurement

Cough severity can be objectively measured in a number of ways. Cough frequency and cough reflex sensitivity are the two most commonly used in research settings, with a growing interest in measuring the force or intensity of coughing. Cough monitors measure cough frequency using an external microphone that records patients' cough for extended periods of ≥24 h. VitaloJAK (Vitalograph Ltd, Buckingham, UK) and the Leicester cough monitor are two validated monitors (70, 71), which have been used for research. Cough reflex sensitivity is assessed by the controlled inhalation of protussive compounds such as capsaicin and citric acid. Capsaicin is the most used agent, with a proven safety profile and predictable and repeatable dose-response (28, 72) in patients with IPF. Measurement of cough reflex sensitivity is often used in early phase studies to prove engagement of a drug molecule with its target, as well as to measure the ability to suppress cough using antitussive therapies (73). It is not yet practical to measure the intensity of cough objectively in clinical settings. Potential techniques include pneumotachography for expiratory airflow measurement, electromyography studies of the respiratory muscles, and measurement of transdiaphragmatic pressures (74), which are yet to be evaluated in patients with IPF. Cough intensity may be particularly relevant in this population, given the suggested pathophysiological role of mechanical stretch in augmenting the activation of pro-fibrotic mechanisms.

### Patient Reported Outcome Measures (PROMs)

Patient reported outcomes (PROs) have gained importance in recognition of their value to capture patients' perceptions of their symptoms and health status. They are measured using standardized questionnaires, which may be generic, disease-specific, or symptom-specific. PROMs for cough have been developed in other conditions such as lung cancer, while no cough-specific instruments have been developed and validated prospectively in the IPF population. The currently used cough questionnaires were developed in patients with chronic cough and other respiratory conditions such as chronic obstructive pulmonary disease. Instead of comprehensively reporting on all PROMs for cough that have been used in IPF, this review focuses on selected commonly used cough-specific instruments, including the VAS, LCQ (75), Cough Quality of Life Questionnaire (CQLQ) (76), and Cough and Sputum Assessment Questionnaire (CASA-Q) (76) (Table 2).

**Table 2:** Summary of measurement properties of PROMs for cough assessment in IPF.

**Measurement tool**	**References**	**Population**	**Psychometric properties**	**Results**
**Cough-specific instruments**				
LCQ	Key et al. (11)	19 participants with IPF	Construct validity	Spearman's correlation coefficients for objective cough frequency and LCQ total *r* −0.80 (*p* < 0.001) and LCQ physical *r* 0.76 (*p* < 0.001)^*^
		DLCO% 43.2 ± 16.06, FVC% 78.5 ± 24.4		
	Morice et al. (77)	45 participants (29 with IPF) TLCO% 49 ± 16	Construct validity	Correlations between LCQ domains and measures of disease severity were weak and non-significant.
			Test-retest reliability	ICCs for total and domain scores ranging from 0.37 to 0.58
VAS	Key et al. (11)	19 participants with IPF DLCO% 43.2 ± 16.06, FVC% 78.5 ± 24.4	Construct validity	Spearman's correlation coefficients for objective cough frequency and VAS for cough severity^*^
				Day cough rate *r* = 0.8 (*p* < 0.001) and night cough rate *r* = 0.71 (*p* = 0.001).
CQLQ	Lechtzi et al. (78)	20 participants with IPF	Internal consistency	Cronbach's a >70 for total score and 4/6 subscales
		DLCO% 57.4 ± 14.4, FVC% 70.4 ± 13.7	Test-retest reliability	ICC for total score at baseline and week 15 0.88 (*p* < 0.01)
			MID	MID was 5.7 (95% CI, 4.9-6.4)
CASA-Q	Gries et al. (79)	18 participants with IPF DLCO% 48.7 ± 15.1, FVC% 87.2 ± 30.7	Content validity	^†^Intended meaning of each item clearly understood (89–100%).
				Items perceived to be highly relevant
				Recall period accurately used 89%.
**Disease-specific instruments with cough domains or items relevant to cough**				
ATAQ-IPF	Horton et al. (80)	95 participants with IPF	Internal consistency	^†^Cronbach's a 0.92, Rasch model reliability 0.83
		DLCO% 39 ± 15, FVC% 65 ± 17,		
ATAQ-IPF-cA	Birring et al. (81)	139 participants with IPF	Construct validity	Two items were removed due to floor effects
		DLCO% 42.2 ± 11.9 – 43.2 ± 16.5,		
		FVC% 70.1 ± 17.7 – 80.7 ± 9.2^‡^	Internal consistency	^†^Internal consistency Cronbach's a 0.92
L-IPF	Swigris et al. (82)	125 participants with IPF DLCO% 50 ± 20, FVC% 71 ± 20	Construct validity	Correlation between L-IPF cough domain and SGRQ symptom domain 0.67. Correlations between L-IPF domain for cough and disease severity significant.
			Internal consistency	^†^Internal consistency ω coefficient > 0.93
			Test-retest reliability	^†^ICC 0.85
SGRQ-I	Prior et al. (83)	150 participants with IPF	Internal consistency	^†^Cronbach's a 0.77
		DLCO% 48.4 ± 14.1, FVC% 87.2 ± 23.1	Test-retest reliability	^†^ICC 0.78

*ATAQ-IPF, A Tool to Assess QOL in IPF; ATAQ-IPF-cA, A Tool to Assess QOL in IPF Cross Atlantic; CASA-Q, Cough and Sputum Assessment Questionnaire; CQLQ, Cough Quality-of-Life Questionnaire; DLCO, diffusing capacity for carbon monoxide; FVC, forced vital capacity; ICC intraclass correlation coefficient; IPF, idiopathic pulmonary fibrosis; LCQ, Leicester Cough Questionnaire; L-IPF, Living with Pulmonary Fibrosis Questionnaire; MID, minimally important difference; SGRQ-I, IPF-specific version of the St George's Respiratory Questionnaire; TLCO, transfer factor for carbon monoxide; VAS, Visual Analog Scale; %, percentage predicted*.

* *Evaluated in IPF*.

† *Results for cough domains quoted*.

‡ *Results expressed as range of means for United Kingdom and United States of America populations*.

*Results expressed as mean ± standard deviation or mean ± standard error*.

#### Cough Severity Visual Analog Scale

VAS subjectively assesses cough severity and is popular due to its simplicity. Patients are asked to place a vertical mark on a 0–100-mm linear scale reflecting their cough intensity or frequency, with higher scores indicating greater severity. It can be used to assess both the status and changes in cough severity. There is limited robust data on its reliability and validity in specific respiratory conditions, including asthma and chronic obstructive pulmonary disease (84). The VAS is a responsive outcome measure in patients with acute cough (85) and when used as an outcome measure in intervention studies of chronic cough (86). Elevated VAS score predicts persistent anxiety and depression in IPF (87). However, in a recent study of patients with ILD, the VAS could not detect a change in cough symptoms (88). Further research is needed to validate VAS for cough measurement in patients with IPF.

#### Leicester Cough Questionnaire

The LCQ is the most widely used validated cough instrument in clinical and research settings. Its advantages are that it is relatively easy to administer, translated into many languages, and used in many cultures. It is a self-administered questionnaire consisting of 19 items covering three health domains (physical, psychological, and social) using a 7-point Likert response scale for each item. The total LCQ score ranges from 3 to 21, with lower scores indicating greater impairment in health status because of cough. All domains of the LCQ inversely correlate with objective daytime cough frequency (11) in patients with IPF. The established minimally important difference for the total LCQ score in patients with chronic cough is 1.3 points (89), which is yet to be determined in the IPF population.

#### Cough Quality of Life Questionnaire

The CQLQ consists of 28 items and six health domains (physical complaints, extreme physical complaints, psychosocial issues, emotional well-being, personal safety fears, and functional abilities). Each item is rated using a 4-point Likert scale. The CQLQ has good internal consistency in IPF but this does not apply to all domains (78). Internal consistency is low for both the extreme physical complaints and the personal safety fears domains. Compared to the LCQ, the CQLQ contains more items related to physical impact of cough and concerns about having a serious undiagnosed underlying cause of cough, the latter of which is irrelevant in patients with an established diagnosis of IPF (78). Early evidence on the test-retest reliability has been positive, albeit at a study interval of 15 weeks (78) as test-retest time intervals commonly range between 2 days and 2 weeks (90). The minimally important difference for the CQLQ total score is estimated between 5 and 5.7 in patients with IPF (78), in contrast with 10.58–21.89 in patients with chronic cough (91).

#### Cough and Sputum Assessment Questionnaire

The CASA-Q comprises four domains: symptom and impact for cough and sputum. The score for each domain ranges from 0 to 100, and lower scores indicate worse symptoms. It has been used in landmark trials for antifibrotic therapies in IPF (48, 92) with well-established content validity (79); however, its reliability and responsiveness in IPF have not yet been demonstrated (93).

### Cough as an Outcome Measure in Clinical Trials

Clinical trial design in IPF has historically been hampered by a lack of consensus on appropriate outcome measures to reliably assess treatment response (94). For example, cough was not identified by the medical expert Delphi process as a core outcome domain for use in clinical trials of patients with IPF and connective tissue disease-associated ILD (94). In contrast, it was nominated as a central symptom by patients in focus groups, reflecting worsening of their condition (94). Recently, cough has been acknowledged and included as a domain in the development of comprehensive health-related quality of life measures for IPF such as the Living with IPF (L-IPF) questionnaire (82) and the IPF-specific version of the St George's Respiratory Questionnaire (83). Objective cough count measured by cough frequency monitors has traditionally been used as the preferred primary endpoint in clinical trials for antitussive medications. Expert consensus recommends the combined use of subjective instruments and objective cough frequency for evaluation of cough (95), as they provide complementary information on the severity and impacts of cough.

## Therapies

There is no therapy for cough in IPF that has proved effective in clinical trials. Aside from treatment of relevant comorbidities, several therapies have been tried for chronic cough in IPF and may be useful in some individuals, which include both non-pharmacological and pharmacological interventions (Table 3). It is suggested that most antitussive interventions are trialed for 2 weeks before re-evaluation and change of therapy. For refractory cough, referral to specialized cough clinics for a trial of speech therapy or physiotherapy and neuromodulation can be considered. Patients with end-stage disease may benefit from referral to specialist palliative care for symptom control.

**Table 3:** Summary of trials of pharmacological therapy in IPF-related cough.

**References**	**Study design**	**Number of participants**	**Intervention**	**Primary endpoint**
Birring et al. (81)	Multi-center randomized double-blind placebo-controlled crossover trial	24	Nebulised sodium cromoglycate (PA101) *via* nebuliser 40 mg tds for 14 days	Mean reduction in daytime objective cough frequency (coughs per hour) by 31.1%
Horton et al. (80)	Randomized, double-blind, placebo-controlled cross over	24	Thalidomide 50–100 mg daily for 12 weeks	Improvement in cough-specific quality of life (CQLQ) (mean difference v. placebo, −11.4 [95% CI, −15.7 to −7.0]; p < 0.001)
Lutherer et al. (96)	Proof of concept, uncontrolled single-arm study	12	IFN-alpha lozenges 150 IU tds for 12 months	Improvement in cough-specific quality of life (LCQ) in 5 of 6 participants^*^
van Manen et al. (26)	Multi-center prospective observational study	43	Pirfenidone dosed according to clinical practice for 12 weeks	Objective 24-h total cough count decreased by 34% [95% CI, −48 to −15] at 12 weeks
Guler et al. (97)	Randomized, double-blind, placebo-controlled cross over	20	Azithromycin 500 mg three times per week for 12 weeks	No significant change in cough-specific quality of life (LCQ)

*CI, confidence interval; CQLQ, Cough Quality of Life Questionnaire; IFN, interferon; IU, international units; LCQ, Leicester Cough Questionnaire; tds, three times per day; VAS, Visual Analog Scale*.

* *Secondary endpoint: improvement in cough-specific quality of life measured in a subgroup of participants with chronic cough at 2–3 weeks*.

### Non-Pharmacological

#### Multimodality Speech Therapy

While the evidence is scarce in patients with IPF, multimodality speech therapy, an established therapy for unexplained chronic cough, may be a suitable treatment. Speech therapy has been reported to decrease cough frequency and improve quality of life in chronic cough (98). The Physiotherapy, Speech and Language Therapy Intervention has shown similar benefits in patients with chronic cough (99). These therapies include education about cough and cough control techniques, laryngeal hygiene, and psychoeducational counseling, which may be used alongside neuromodulator medications for cough reflex sensitivity such as gabapentin or pregabalin.

### Pharmacological

#### Antifibrotic Drug Therapies

The outlook for patients with IPF changed with the availability of two antifibrotic drug therapies; nintedanib and pirfenidone. Nintedanib alters cell signaling mediated by tyrosine kinases resulting in the inhibition of mechanisms fundamental to the development of fibrosis (100). Pirfenidone exhibits pleiotropic antifibrotic, anti-inflammatory and antioxidant properties, which has recently been linked to the inhibition of glioma-associated oncogene homolog transcription factors (101). These drugs have been approved for clinical use by regulators based on their proven efficacy in slowing lung function decline. Animal studies show that pirfenidone decreases cough reflex sensitivity to capsaicin (102). In an observational study of patients with IPF and chronic cough (defined as daily cough over 8 weeks duration and with a cough score of ≥40 mm on a 0–100 mm VAS), pirfenidone reduced objective cough frequency and subjective cough scores measured by the LCQ and VAS at 12 weeks (26). Nintedanib does not improve cough related quality of life or symptom scores as measured by the Living with Pulmonary Fibrosis Questionnaire (103) and CASA-Q (93). Further research is needed to evaluate the therapeutic effects of existing and in-development antifibrotic drugs in future clinical trials, with cough being included as an outcome measure and assessed using validated instruments.

#### Other Pharmacological Therapies

Studies evaluating antitussive therapies in IPF are limited. Opioids have been shown to decrease cough frequency and improve cough-related quality of life in patients with chronic cough in a study of slow-release oral morphine at a dose of 5 mg twice daily (77). Common side effects are constipation and drowsiness, which do not usually require drug cessation. Until further evidence is available, the recommended use of low dose morphine is restricted to advanced IPF where intractable cough impairs quality of life (63). A randomized controlled trial of low dose slow-release morphine in patients with IPF and chronic cough (NCT04429516) is currently underway.

In a randomized placebo-controlled trial of 24 patients with IPF, low-dose thalidomide was shown to improve cough severity and respiratory-related quality of life measured by the CQLQ and VAS scores (80). However, 77% of patients in the treatment arm experienced drug-related adverse effects compared to 22% in the placebo arm. Hence, widespread uptake for its clinical use is limited by the unfavorable side effects, including gastrointestinal disturbance, bradycardia, thromboembolism, peripheral neuropathy, and potential for teratogenicity. Nevertheless, thalidomide may be considered if other agents are contraindicated or have failed. A phase II proof-of-concept trial showed that a novel formulation of sodium cromoglycate (PA101) given *via* high-efficiency nebuliser reduced mean daytime cough frequency by 31% at 14 days in patients with IPF and was generally well-tolerated (81). This benefit has been shown in cough caused by angiotensin-converting enzyme inhibitors and non-small cell lung cancer (104). An older study of six patients with IPF showed therapeutic benefits of high-dose oral corticosteroids for cough (29). However, the detrimental effects of high-dose corticosteroids in IPF have since been well-described (105), and its use for this indication is now discouraged. Low-dose corticosteroids have been tried with little data to support their effectiveness. Several novel drug targets have been identified in unexplained chronic cough, including antagonists at the P2X3 sensory nerve ion channel. A randomized, double-blind, placebo-controlled trial of a P2X3 antagonist, Gefapixant, in patients with IPF and persistent cough showed initial promise, however, the primary endpoint of change in awake objective cough frequency (coughs per hour) was not met (106). Further clinical trials of novel agents for cough in IPF are underway (Table 4).

**Table 4:** Clinical trials under recruitment for cough in IPF.

**NCT trial ID**	**Study name**	**Target recruitment number**	**Study duration**	**Primary endpoints**	Projected end date^*^
NCT04318704	An Open-Label Study of the Efficacy, Safety and Tolerability of NP-120 on Idiopathic Pulmonary Fibrosis and Its Associated Cough	20	14 months	1. ≥50% reduction in the average number of coughs per hour at 12 weeks compared to baseline using an ambulatory cough monitor	September 2021
				2. No worsening of FVC in either mL or % predicted at 12 weeks compared to baseline	
NCT04030026	Phase 2, Double-blind, Randomized, Placebo-controlled, Two-Treatment, Two-Period Crossover Efficacy and Safety Study in Idiopathic Pulmonary Fibrosis (IPF) With Nalbuphine ER Tablets for the Treatment of Cough	60	16 months	Mean daytime cough frequency (coughs per hour) at day 22 using objective digital cough monitoring	December 2021
NCT04429516	PAciFy Cough: A Multicentre, Double-Blind, Placebo-Controlled, Crossover Trial of Morphine Sulfate for the Treatment of PulmonAry Fibrosis Cough	44	20 months	The percentage change in daytime cough frequency (coughs per hour) from baseline as assessed by objective digital cough monitoring at Day 14 of treatment	August 2022

*FVC, forced vital capacity; IPF, idiopathic pulmonary fibrosis; NCT, National Clinical Trial; NP-120, Ifenprodil*.

* *Data obtained from a search of the National Institutes of Health (NIH) and National Library of Medicine (NLM) Clinical Trials Database (ClinicalTrials.gov) on 15th June 2021*.

## Conclusion

Cough is a common and often under-recognized symptom of IPF, which is associated with disease progression. Identified as a central symptom by patients and their caregivers, cough impacts health-related quality of life. Careful and timely clinical evaluation and assessment using validated tools that objectively and subjectively measure cough in this specific patient population are recommended. Currently available measurement tools for chronic cough have been suboptimally validated in patients with IPF. The development of disease-specific PROMs for cough in the IPF population is required to accurately and reliably show the difference between competing therapies. Further clinical research into effective non-pharmacological and pharmacological interventions for cough is vital to improve symptom management and health outcomes for patients with this progressive disease. In light of the limited proven efficacy of single therapeutic agents for cough in IPF, future clinical trials may consider multimodal interventions or combination drug therapy in this patient population.

## Author Contributions

YK and JM conceived the presented idea. JM performed the literature search and wrote the manuscript with support and critical feedback from YK. Critical feedback was also provided by NG and AH. All authors contributed to the final manuscript.

## Conflict of Interest

YK reports grants and personal fees from Boehringer Ingelheim, personal fees from Roche, other from Air Liquide Healthcare, outside the submitted work. NG reports personal fees from Boehringer Ingelheim, Roche, AstraZenaca, and Novartis, other from Air Liquide Healthcare, outside the submitted work. AH reports non-financial support from BOC Ltd Australia and Air Liquide Healthcare, outside the submitted work. The remaining author declares that the research was conducted in the absence of any commercial or financial relationships that could be construed as a potential conflict of interest.

## Publisher's Note

All claims expressed in this article are solely those of the authors and do not necessarily represent those of their affiliated organizations, or those of the publisher, the editors and the reviewers. Any product that may be evaluated in this article, or claim that may be made by its manufacturer, is not guaranteed or endorsed by the publisher.

## Abbreviations

ATAQ-IPF-cA: A Tool to Assess QOL in IPF Cross Atlantic
CASA-Q: Cough and Sputum Assessment Questionnaire
CQLQ: Cough Quality of Life Questionnaire
CPAP: Continuous positive airway pressure
FVC: Forced vital capacity
GOR: Gastroesophageal reflux
ICC: Intraclass correlation coefficient
L-IPF: Living with IPF Questionnaire
ILD: Interstitial lung disease
IFN: Interferon
IPF: Idiopathic pulmonary fibrosis
LCQ: Leicester Cough Questionnaire
MID: Minimally important difference
MUC5B: Mucin 5B
NGF: Nerve growth factor
OSA: Obstructive sleep apnoea
PRO: Patient reported outcome
PROM: Patient reported outcome measure
SGRQ-I: IPF-specific version of St George's Respiratory Questionnaire
TL_CO_: Transfer factor for carbon monoxide
VAS: Visual analog scale.
